# Supplementary material for: Picky with peakpicking: assessing chromatographic peak quality with simple metrics in metabolomics
Source: BMC Bioinformatics. 2023 Oct 28;24:404. doi: 10.1186/s12859-023-05533-4 (PMC10612323; doi:10.1186/s12859-023-05533-4)
Supplement: Supplementary file 2 — Additional file 2: Figure S1 Distribution and single-parameter logistic curves for each metric extracted for model training, shown separately for the MESOSCOPE and Falkor datasets. Histograms show the distribution of good and bad mass features by color across the span of the data on the x-axis with the number of MFs in each bin shown on the y-axis. Scatterplots show the same x-axis but show the results of a logistic regression on the single parameter, with the line of best fit in black and a ± 1 standard error ribbon around it in grey. Vertical jittering has been applied when plotting to reduce the number of overlapping points. [file 12859_2023_5533_MOESM2_ESM.pdf]

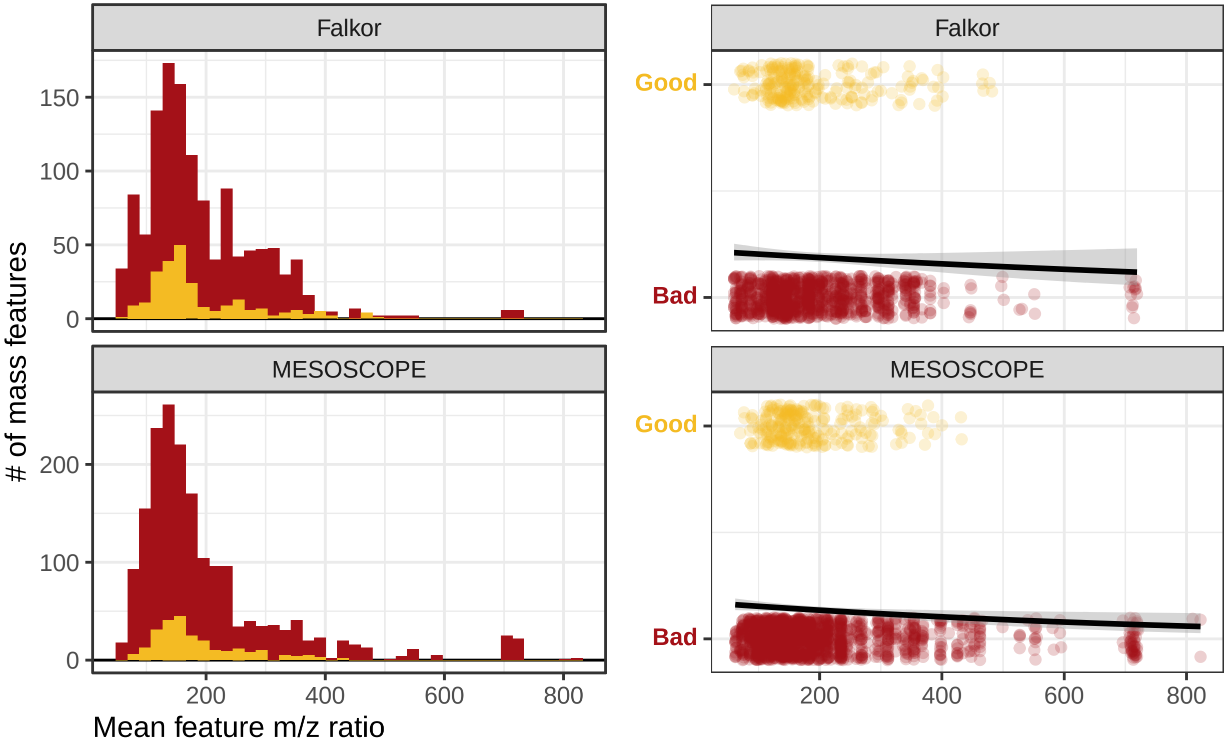

Falkor
MESOSCOPE
Falkor
MESOSCOPE
# of mass features
Mean feature m/z ratio
150
100
50
0
200
100
0
200
400
600
800
Good
Bad
Good
Bad
200
400
600
800

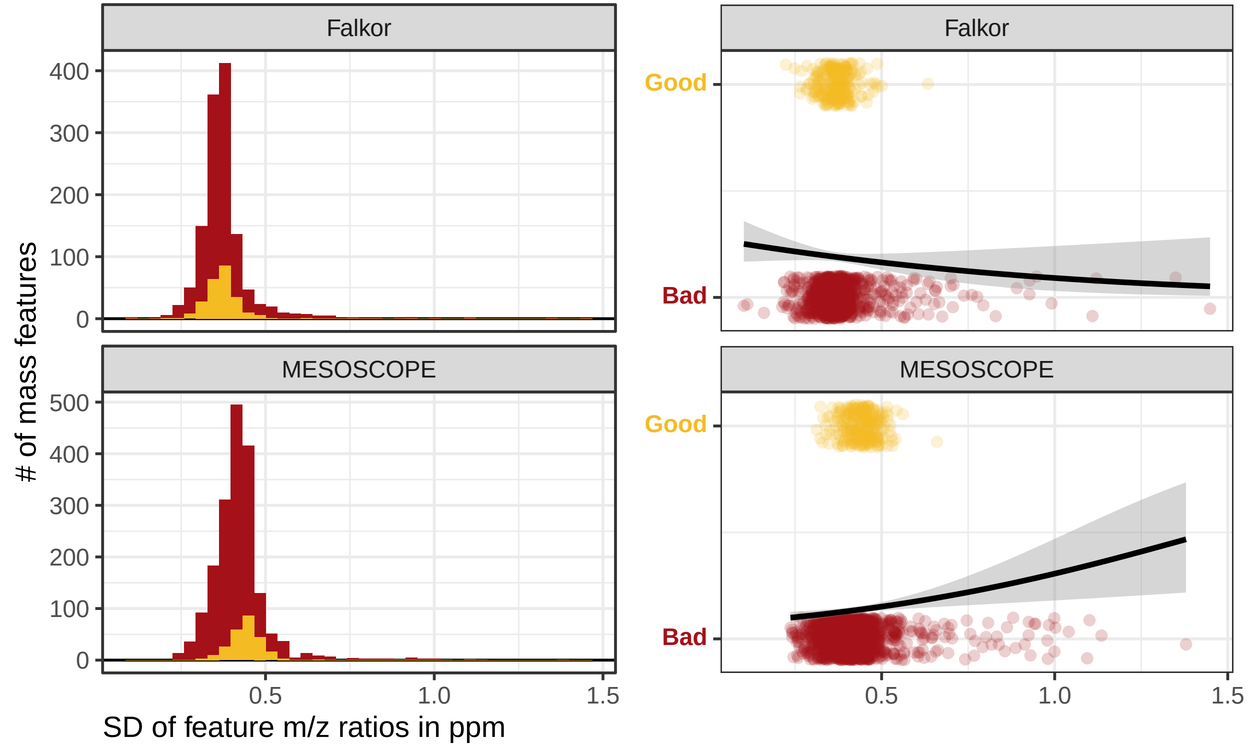
Falkor
MESOSCOPE
# of mass features
SD of feature m/z ratios in ppm
Good
Bad
0
100
200
300
400
500
0.5
1.0
1.5

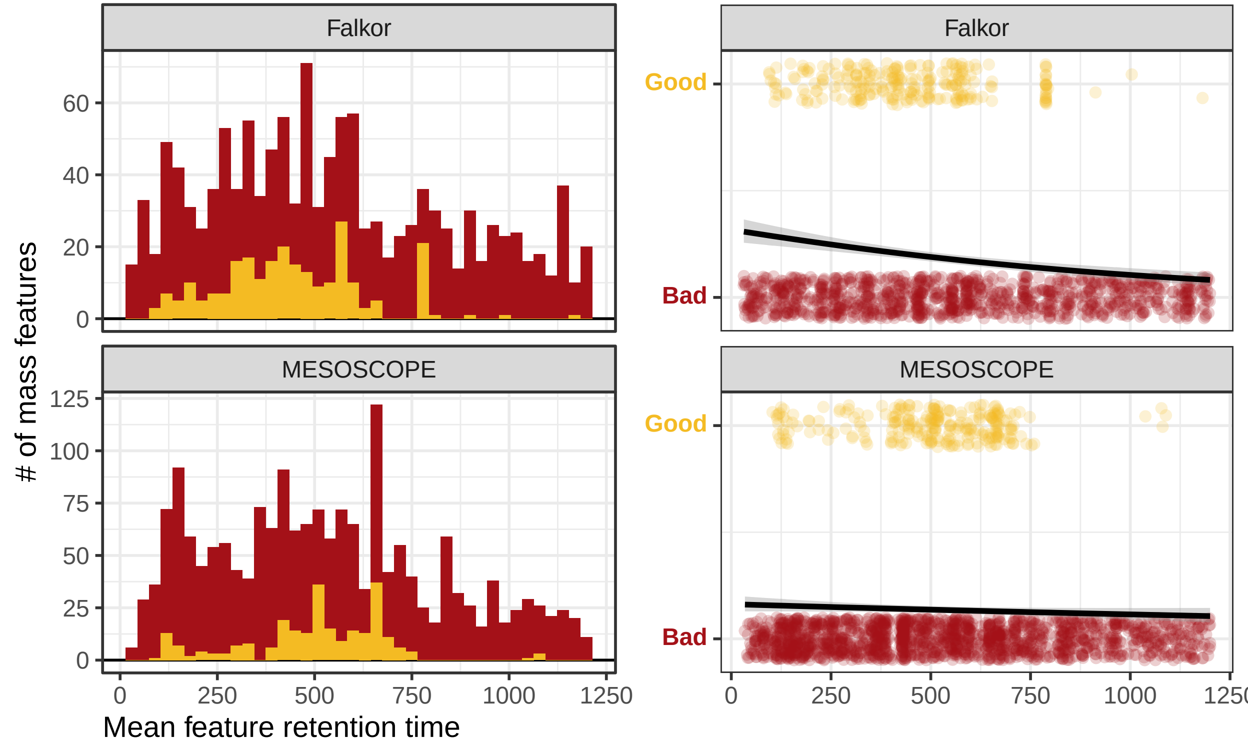
Falkor
MESOSCOPE
# of mass features
Mean feature retention time
Good
Bad
0
20
40
60
25
50
75
100
125
250
500
750
1000
1250

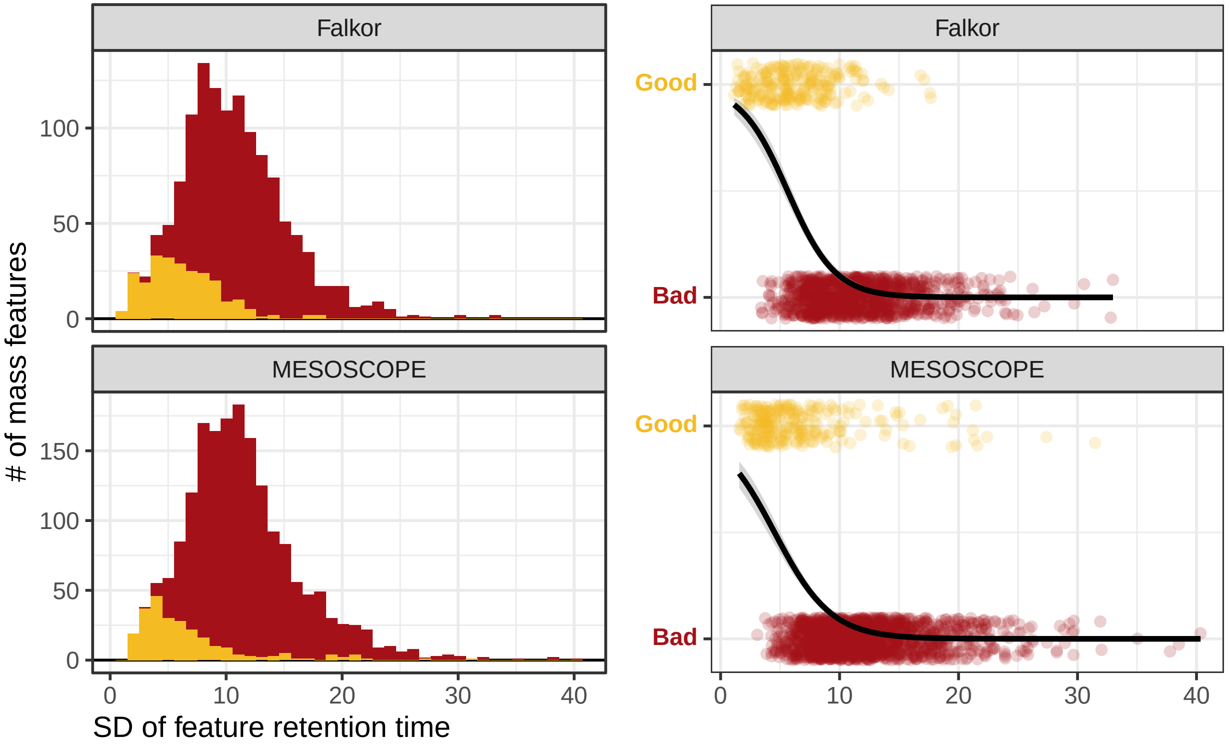

Falkor
MESOSCOPE
# of mass features
SD of feature retention time
0
50
100
150
10
20
30
40
Good
Bad

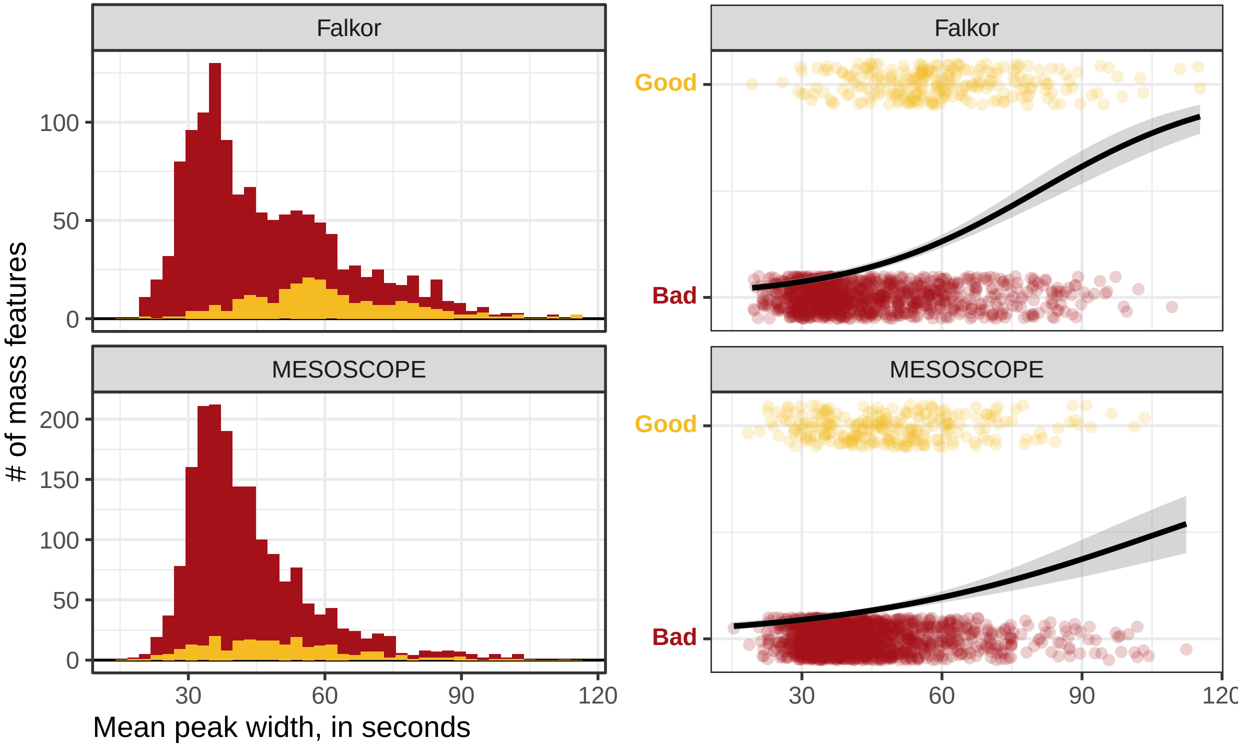
Falkor
MESOSCOPE
# of mass features
0
50
100
150
200
30
60
90
120
Mean peak width, in seconds
Good
Bad

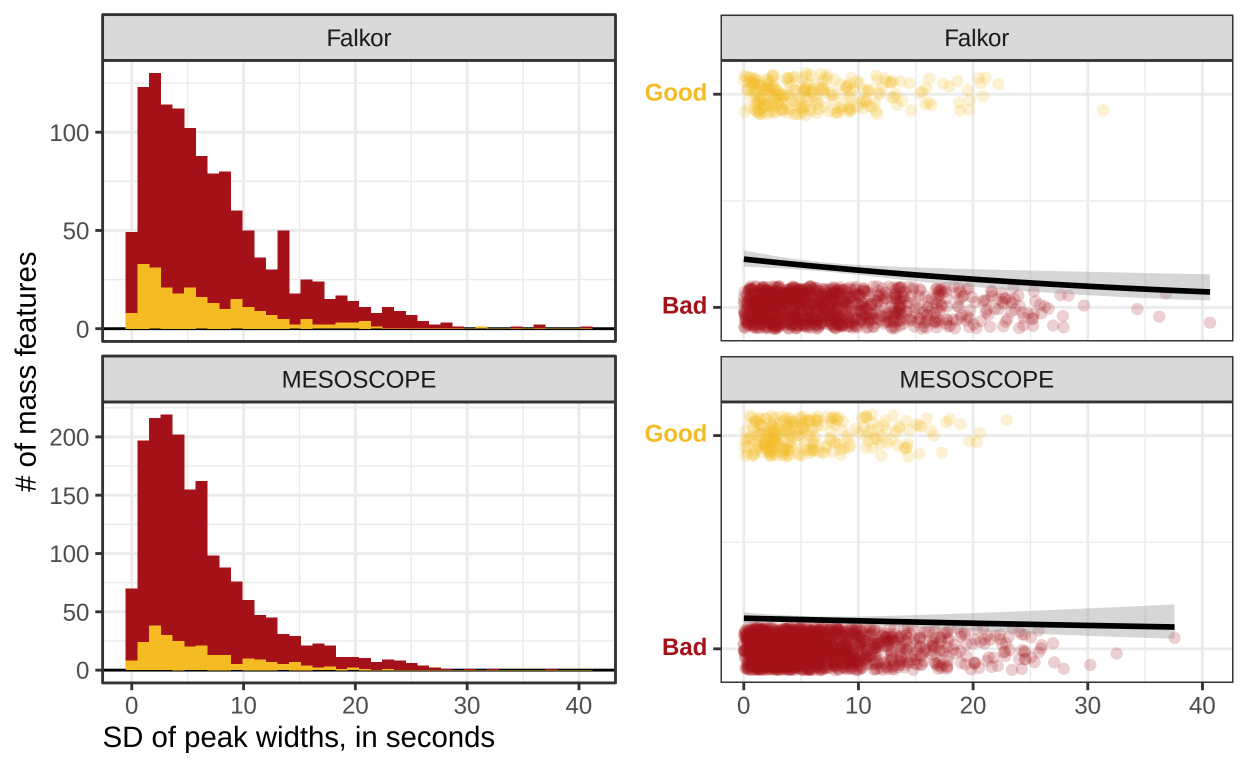

Falkor
MESOSCOPE
# of mass features
SD of peak widths, in seconds
Good
Bad
0
50
100
150
200
10
20
30
40

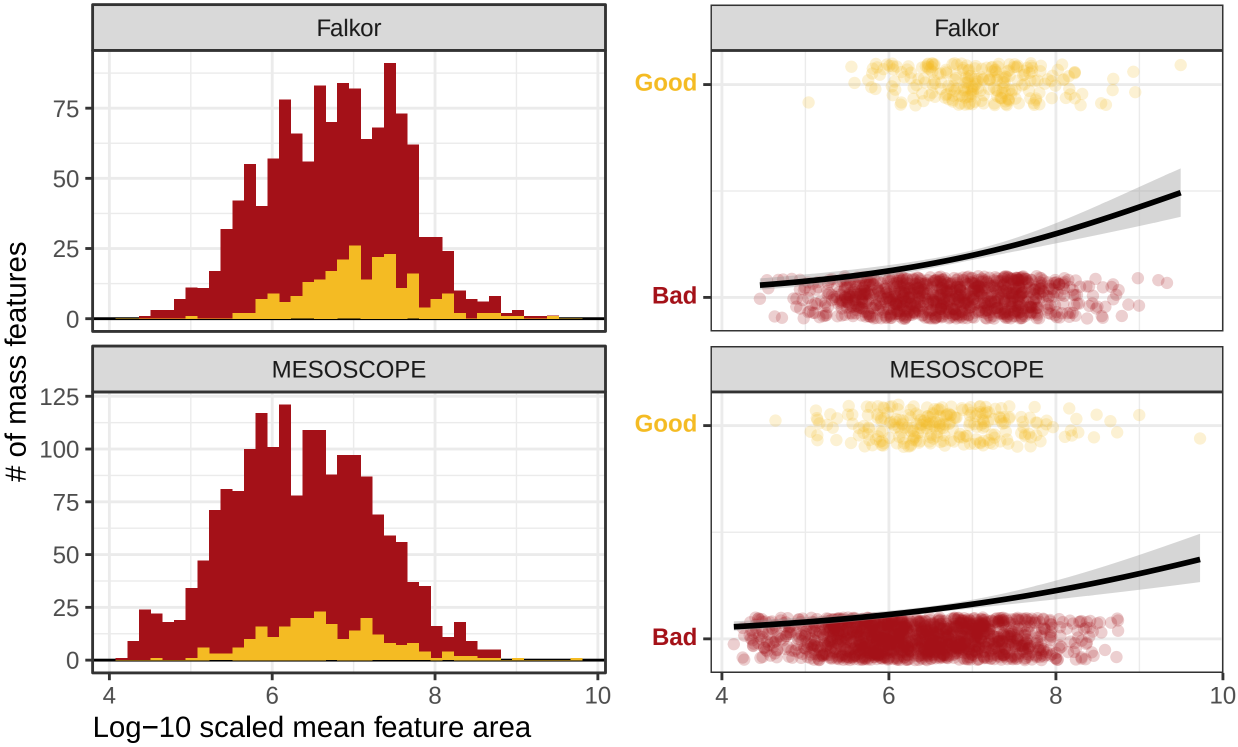

Falkor
MESOSCOPE
# of mass features
Log−10 scaled mean feature area
0
25
50
75
100
125
4
6
8
10
Good
Bad

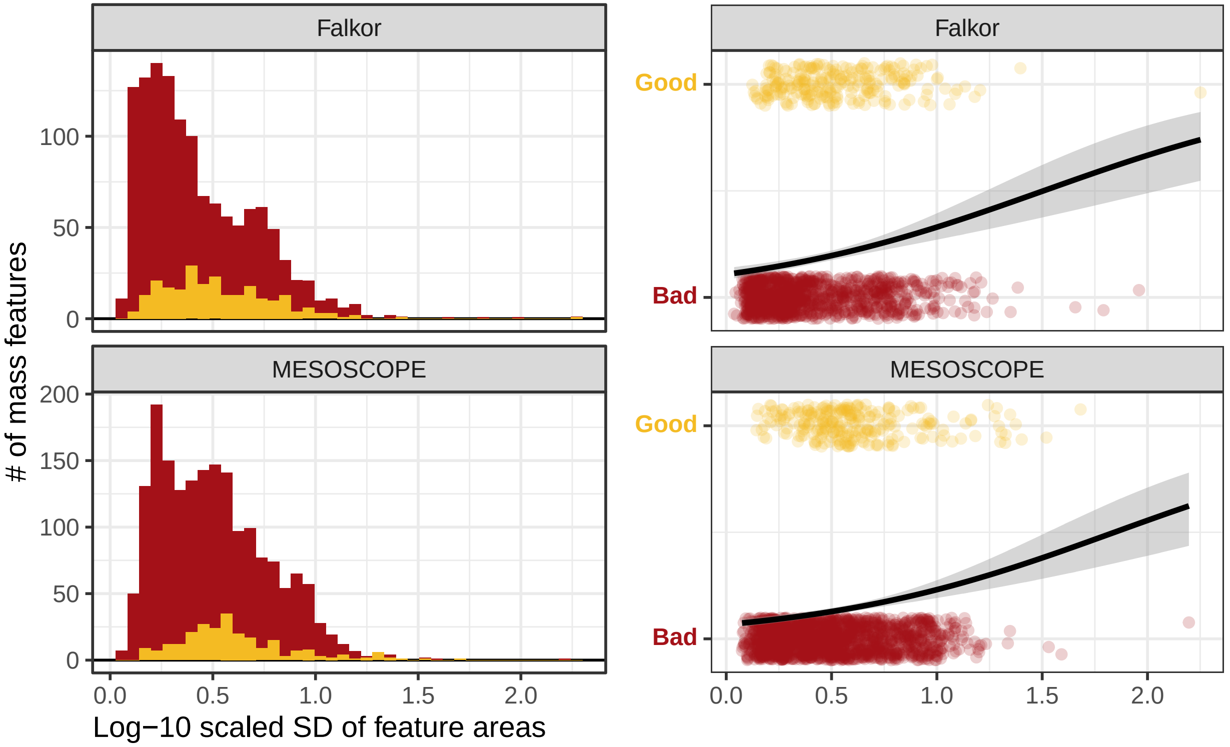
Falkor
MESOSCOPE
# of mass features
Log−10 scaled SD of feature areas
0
50
100
150
200
0.0
0.5
1.0
1.5
2.0
Good
Bad

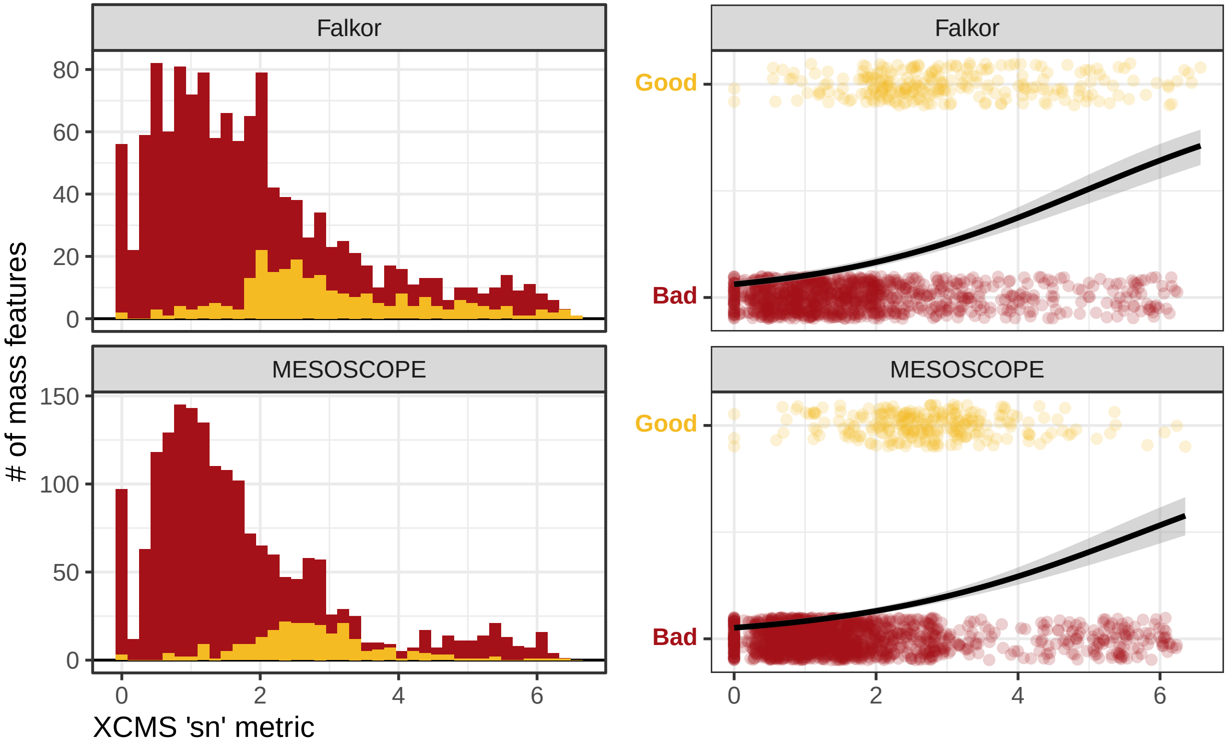
Falkor
MESOSCOPE
Good
Bad
# of mass features
XCMS 'sn' metric
0
2
4
6
80
60
40
20
150
100
50

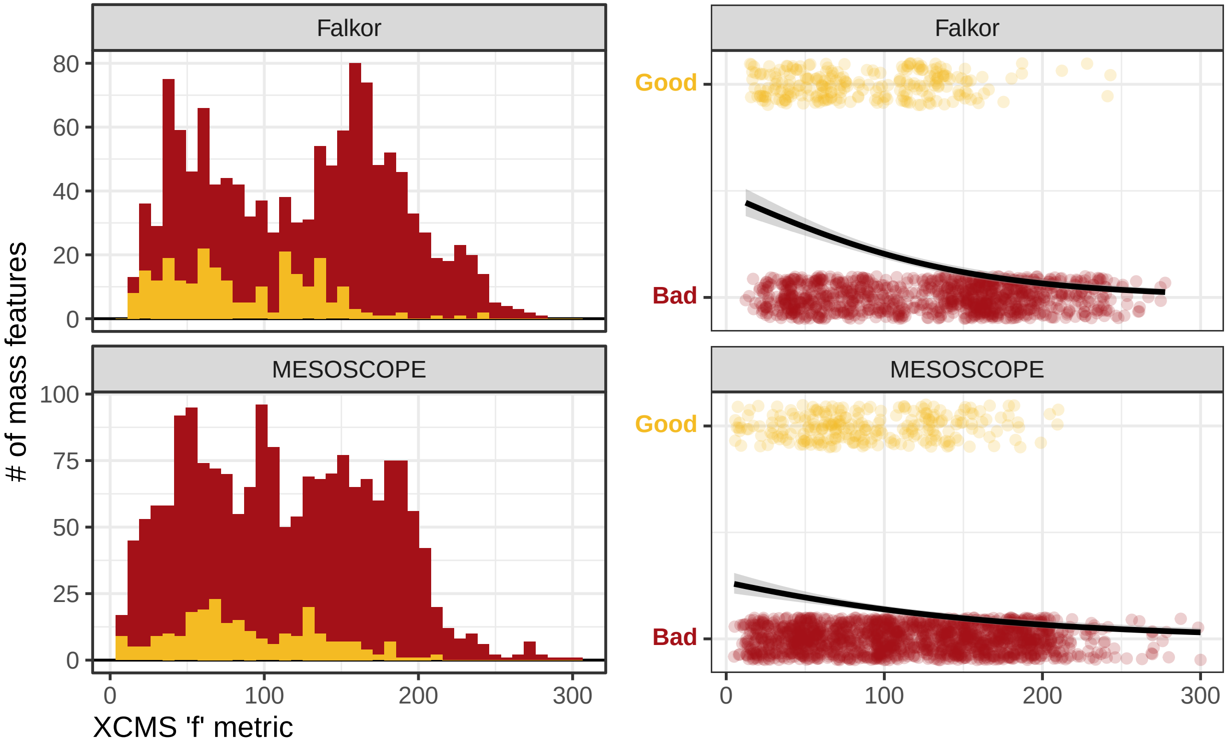

Falkor
MESOSCOPE
Falkor
MESOSCOPE
# of mass features
80
60
40
20
0
100
75
50
25
0
0
100
200
300
XCMS 'f' metric
Good
Bad
Good
Bad
0
100
200
300

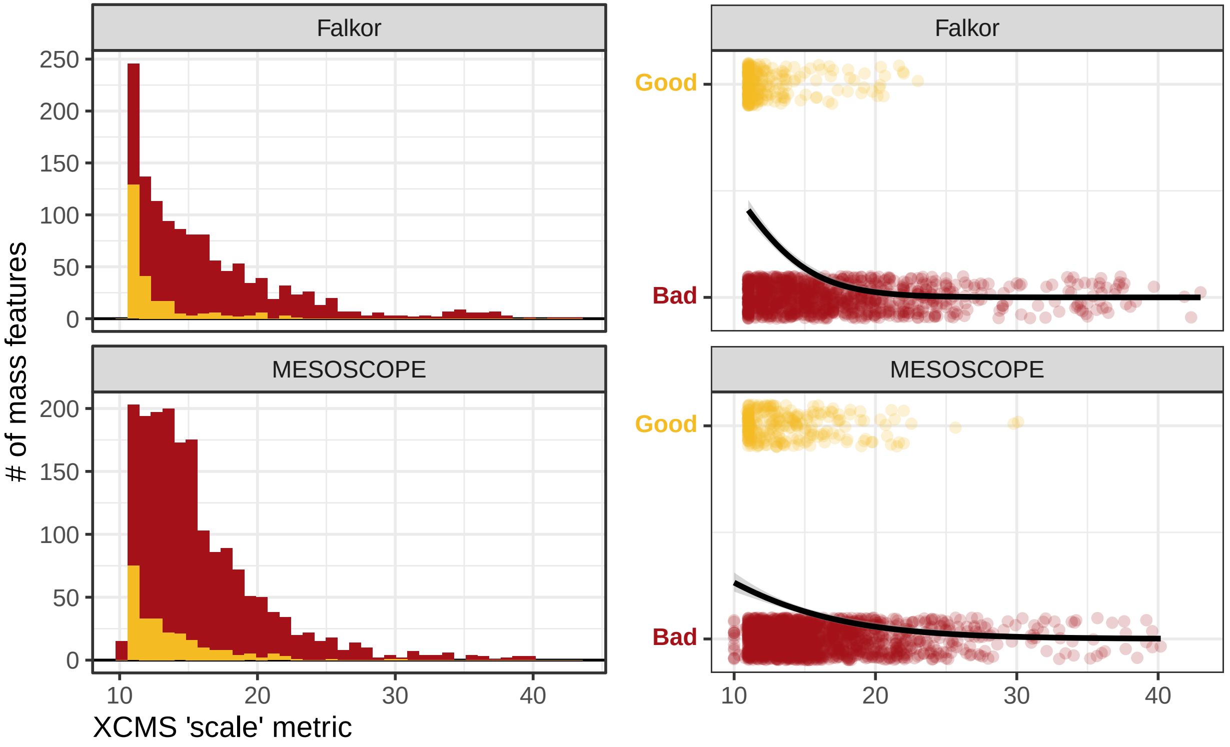

Falkor
MESOSCOPE
Falkor
MESOSCOPE
# of mass features
XCMS 'scale' metric
Good
Bad
Good
Bad
250
200
150
100
50
0
200
150
100
50
0
10
20
30
40
10
20
30
40

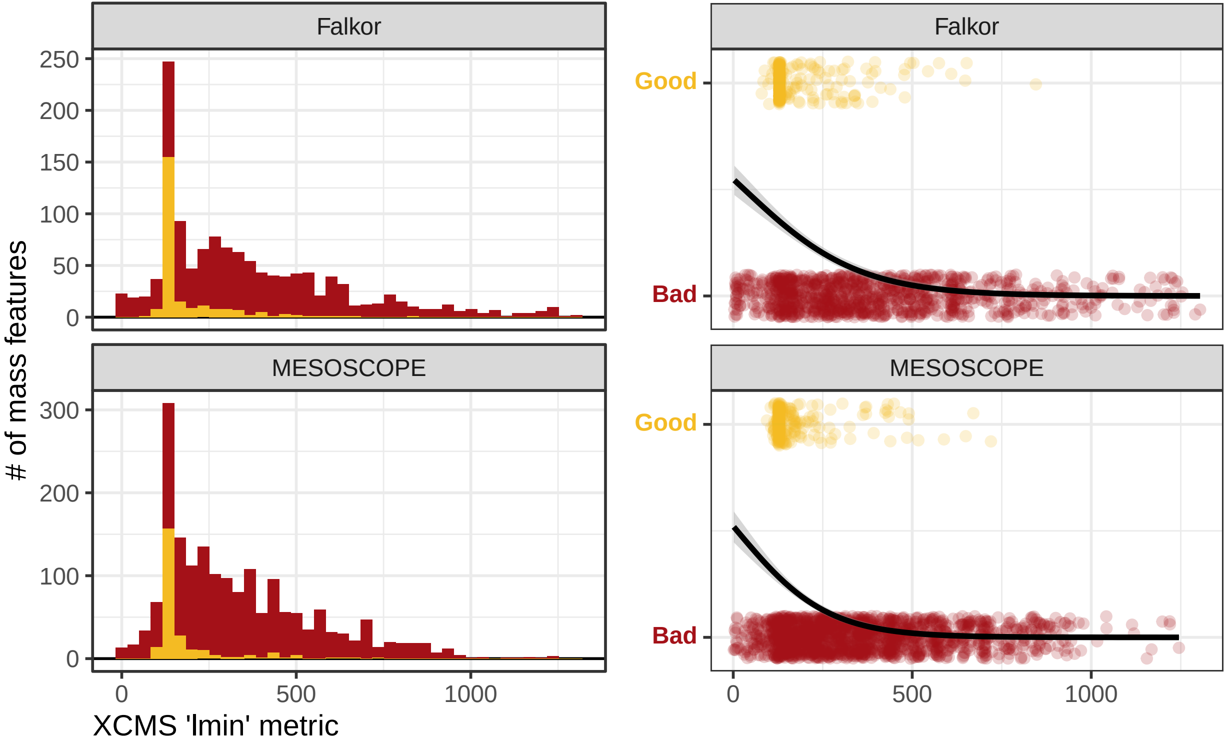

Falkor
MESOSCOPE
# of mass features
250
200
150
100
50
0
300
200
100
0
0
500
1000
XCMS 'lmin' metric
Good
Bad

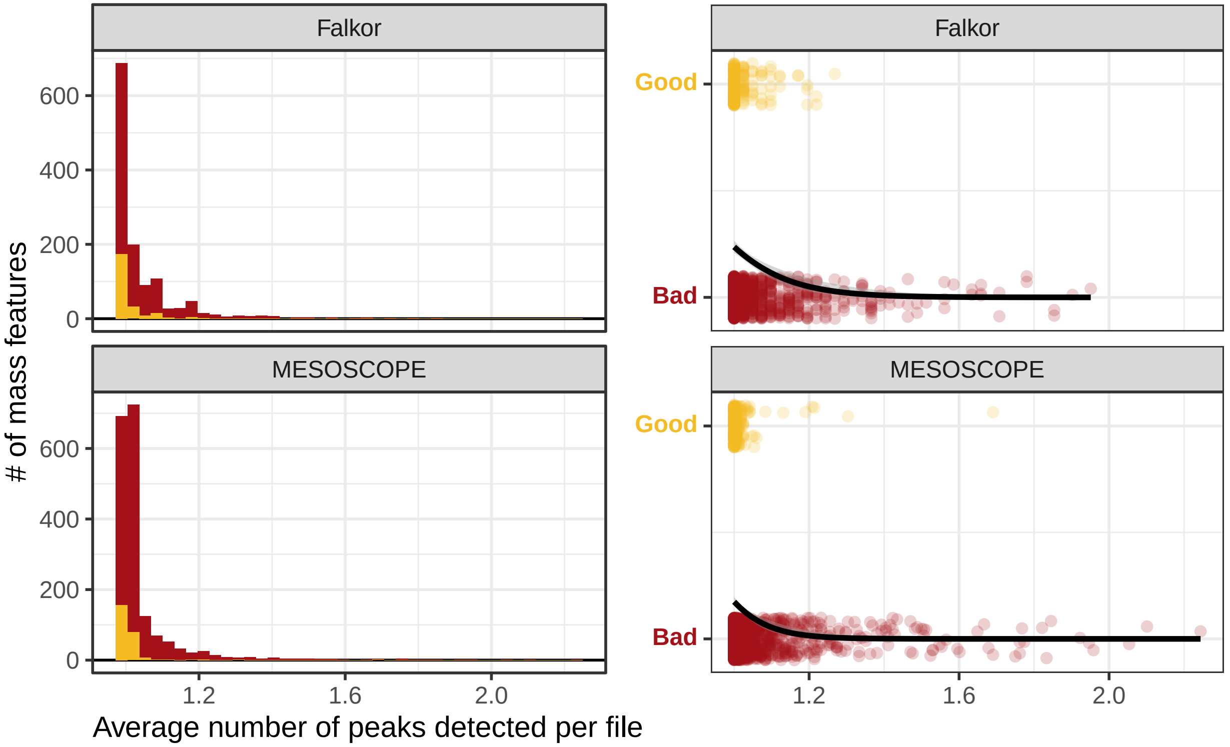

Falkor
MESOSCOPE
# of mass features
0
200
400
600
1.2
1.6
2.0
Average number of peaks detected per file
Falkor
MESOSCOPE
Good
Bad
Good
Bad
1.2
1.6
2.0

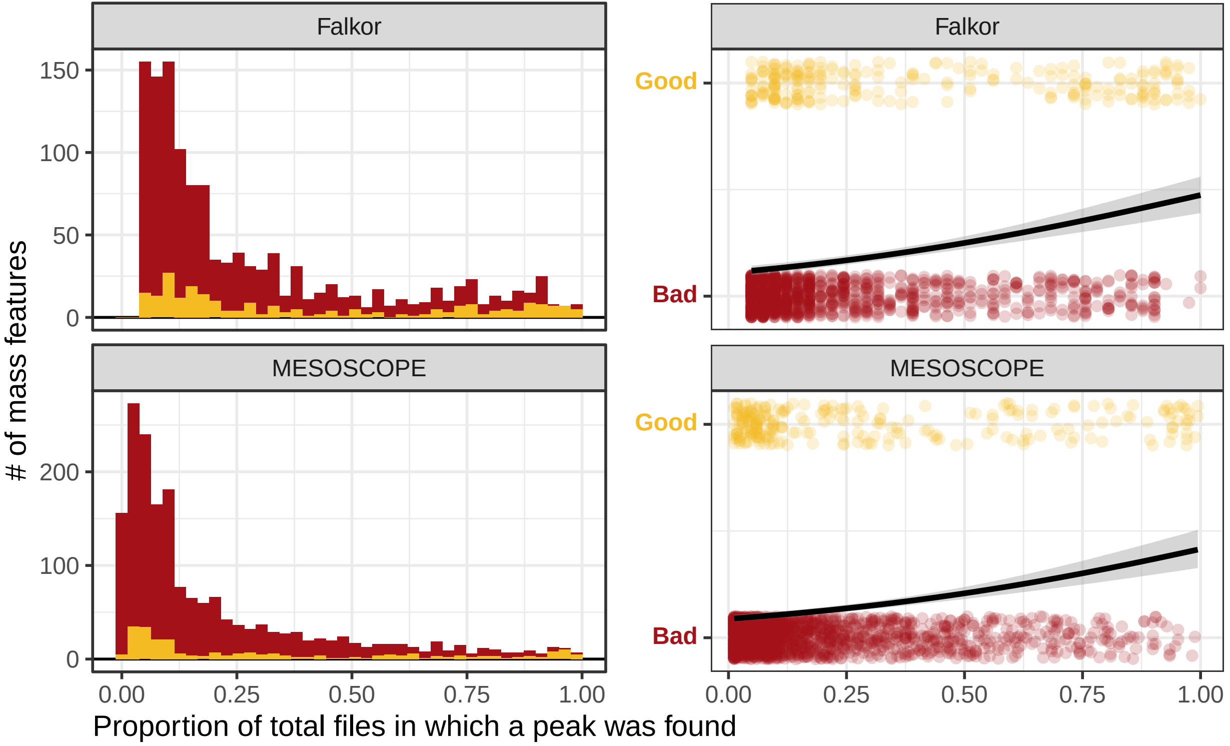

Falkor
MESOSCOPE
Falkor
MESOSCOPE
# of mass features
150
100
50
0
200
100
0
Good
Bad
Good
Bad
0.00
0.25
0.50
0.75
1.00
0.00
0.25
0.50
0.75
1.00
Proportion of total files in which a peak was found

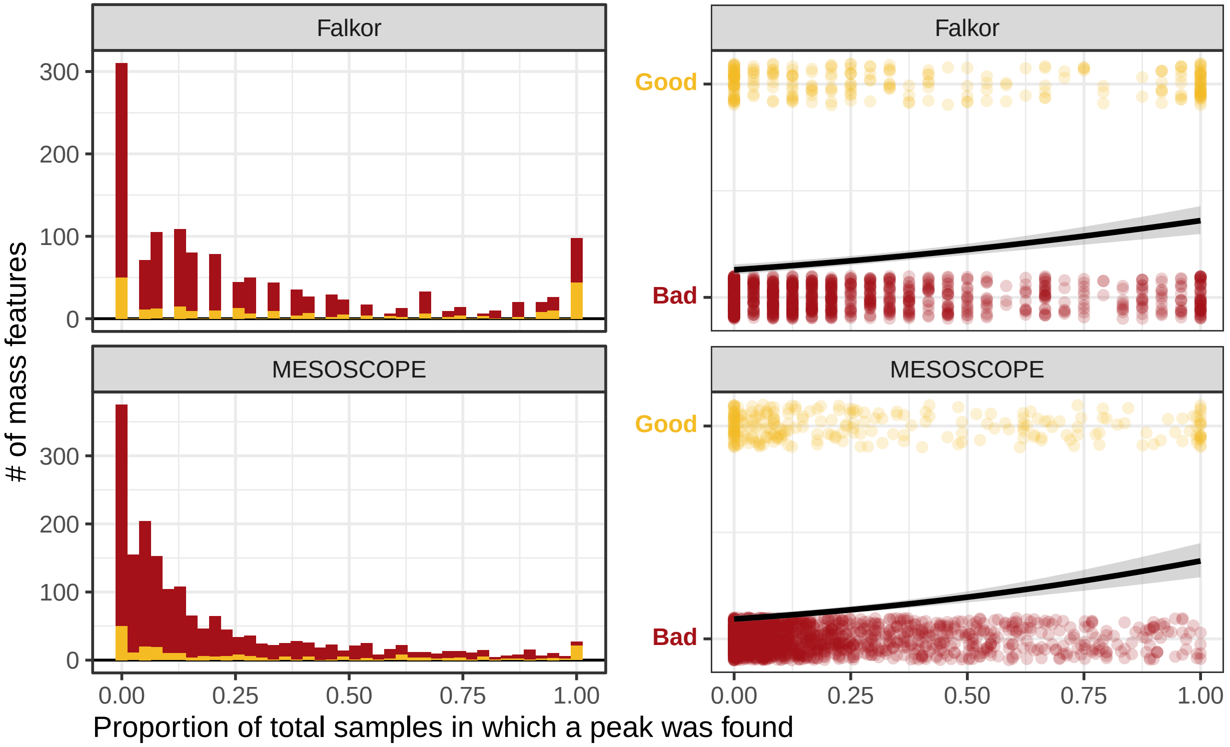

Falkor
MESOSCOPE
Falkor
MESOSCOPE
# of mass features
300
200
100
0
Good
Bad
0.00
0.25
0.50
0.75
1.00
Proportion of total samples in which a peak was found

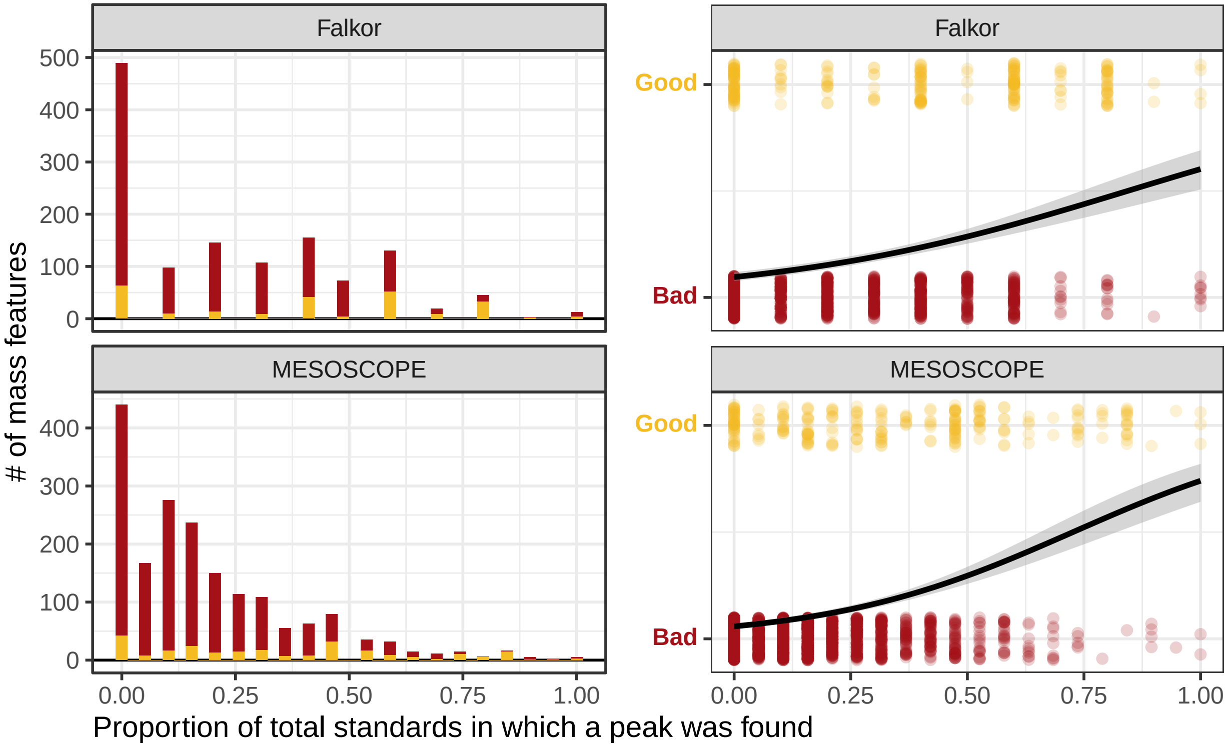
Falkor
MESOSCOPE
# of mass features
500
400
300
200
100
0
0.00
0.25
0.50
0.75
1.00
Good
Bad
Proportion of total standards in which a peak was found

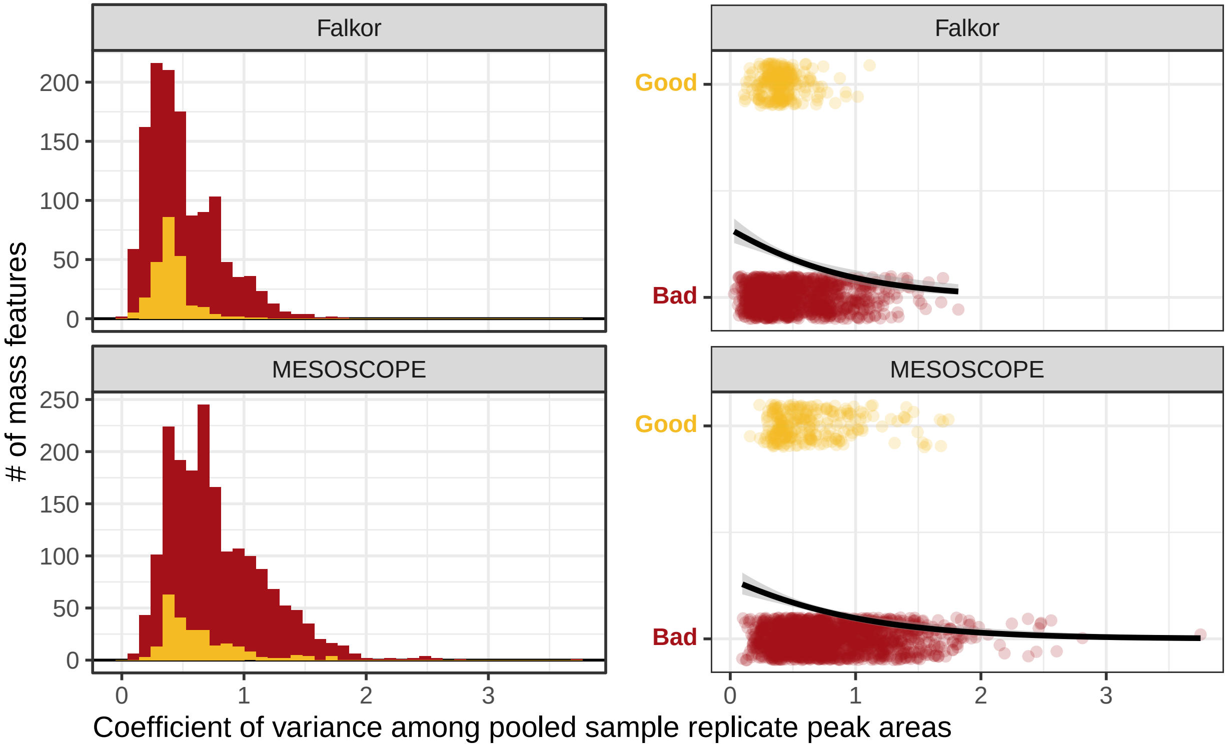
Falkor
MESOSCOPE
Falkor
MESOSCOPE
Good
Bad
Good
Bad
200
150
100
50
0
250
200
150
100
50
0
0
1
2
3
# of mass features
Coefficient of variance among pooled sample replicate peak areas

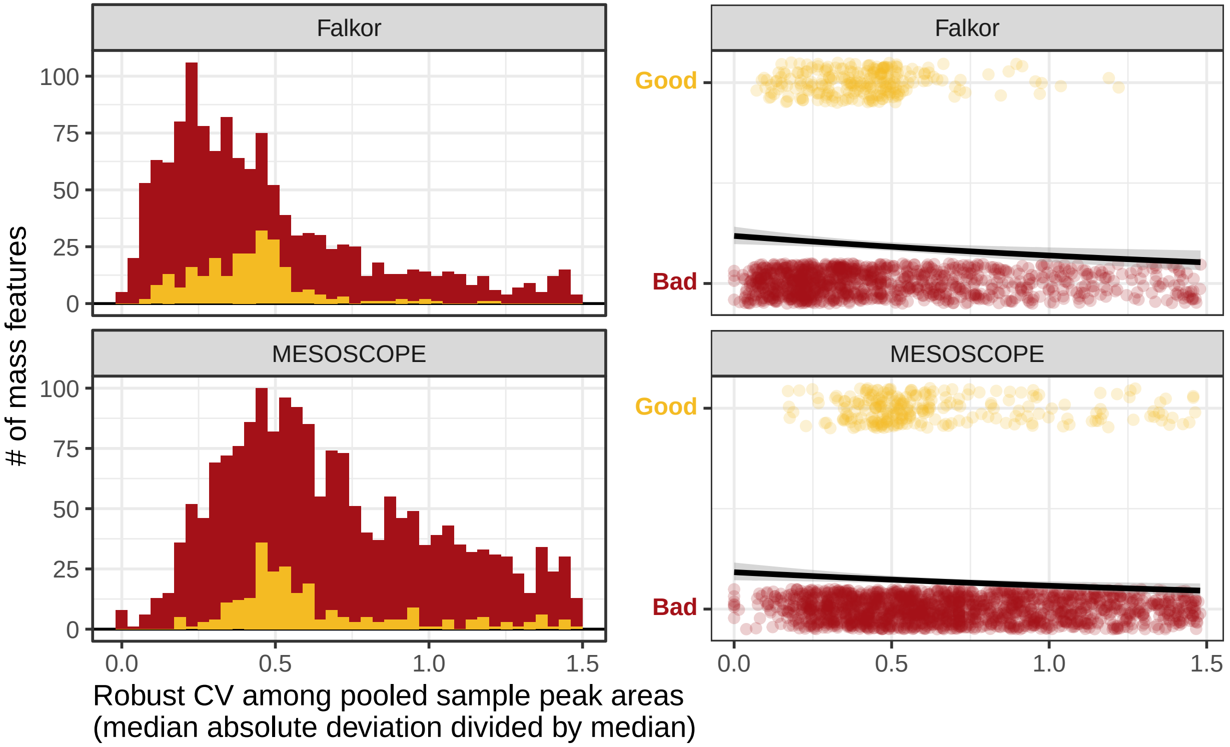

Falkor
MESOSCOPE
# of mass features
100
75
50
25
0
0.0
0.5
1.0
1.5
Good
Bad
Robust CV among pooled sample peak areas
(median absolute deviation divided by median)

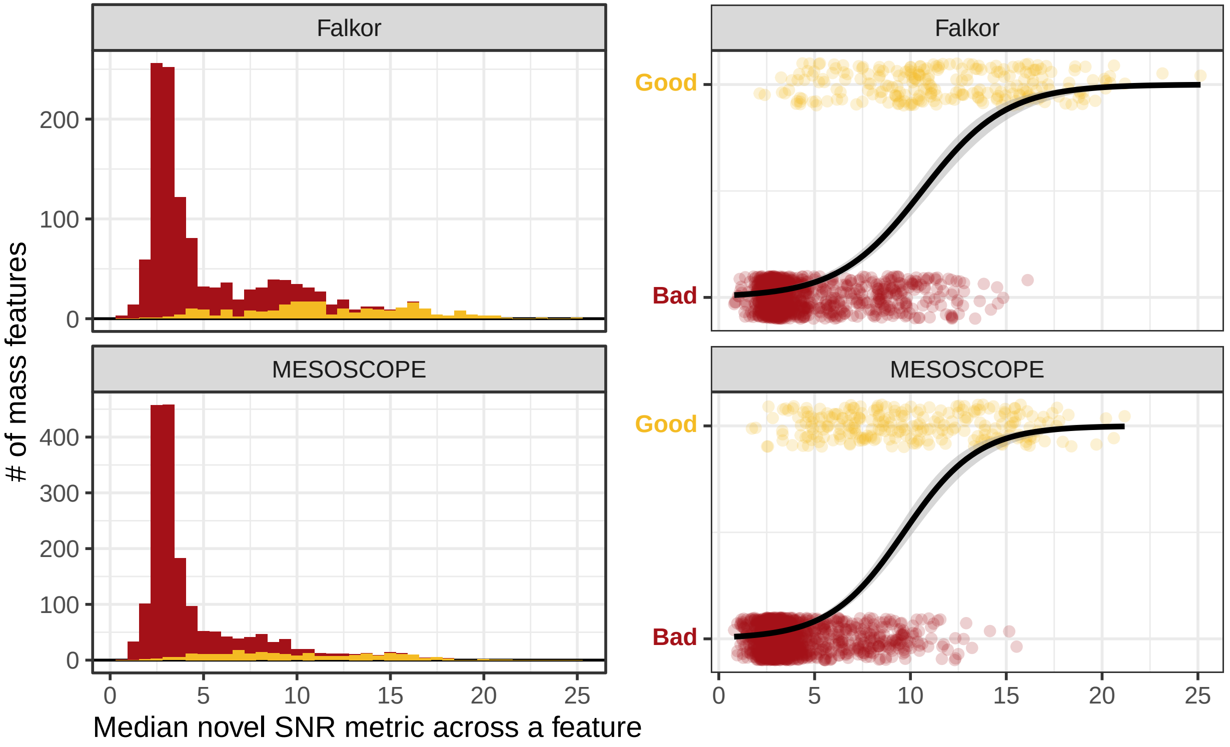

Falkor
MESOSCOPE
# of mass features
Median novel SNR metric across a feature
Good
Bad
0
100
200
300
400
5
10
15
20
25

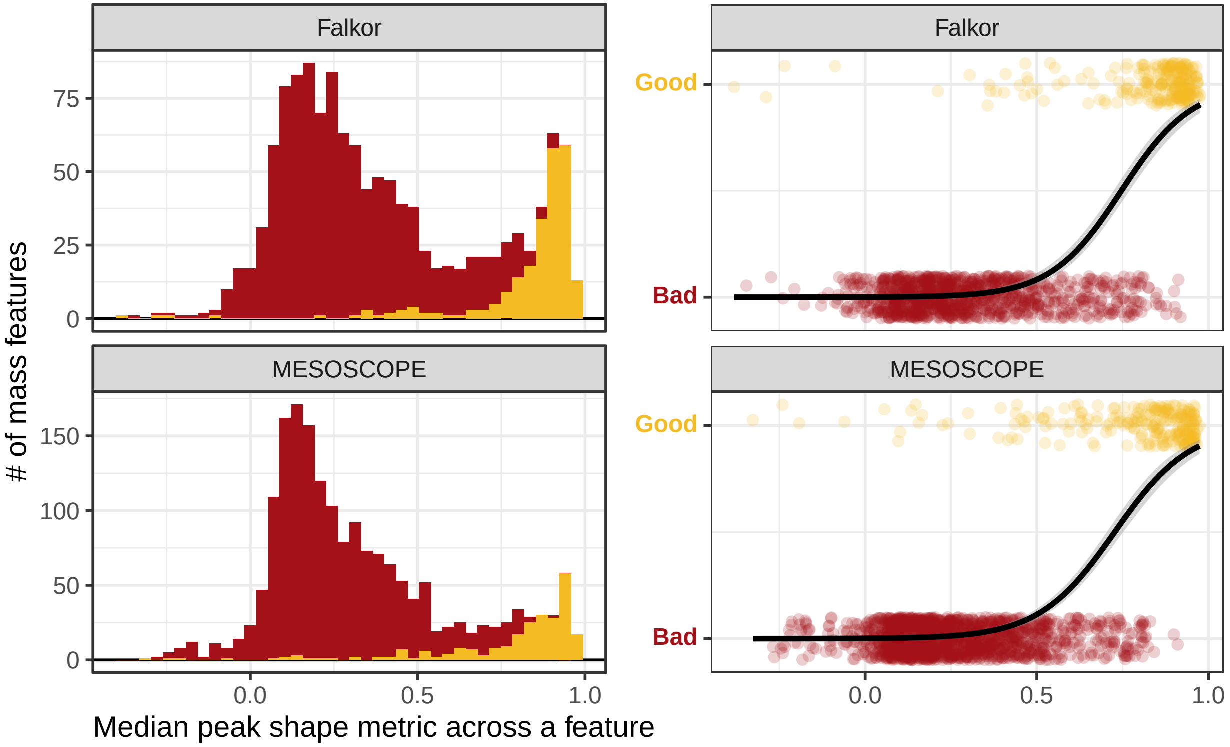

Falkor
MESOSCOPE
Falkor
MESOSCOPE
Good
Bad
Good
Bad
75
50
25
0
150
100
50
0
0.0
0.5
1.0
0.0
0.5
1.0
# of mass features
Median peak shape metric across a feature

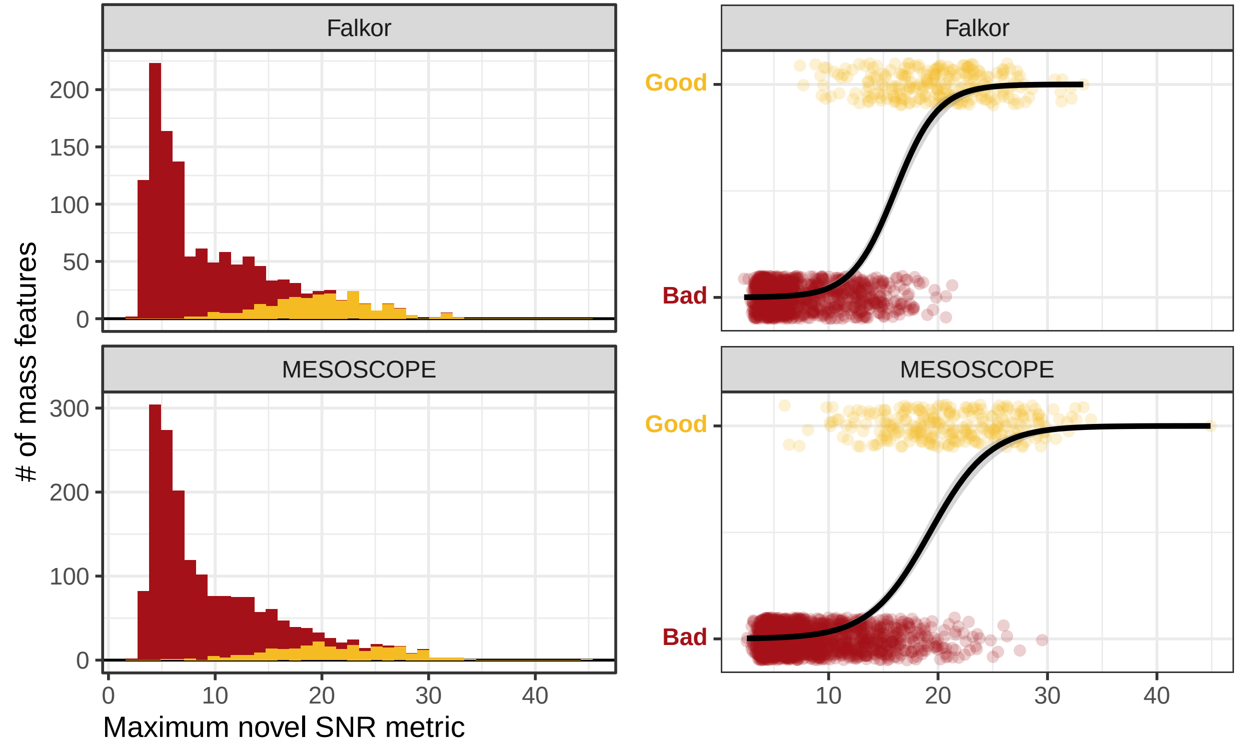
Falkor
MESOSCOPE
Falkor
MESOSCOPE
# of mass features
Maximum novel SNR metric
Good
Bad
Good
Bad
0
50
100
150
200
0
100
200
300
0
10
20
30
40
10
20
30
40

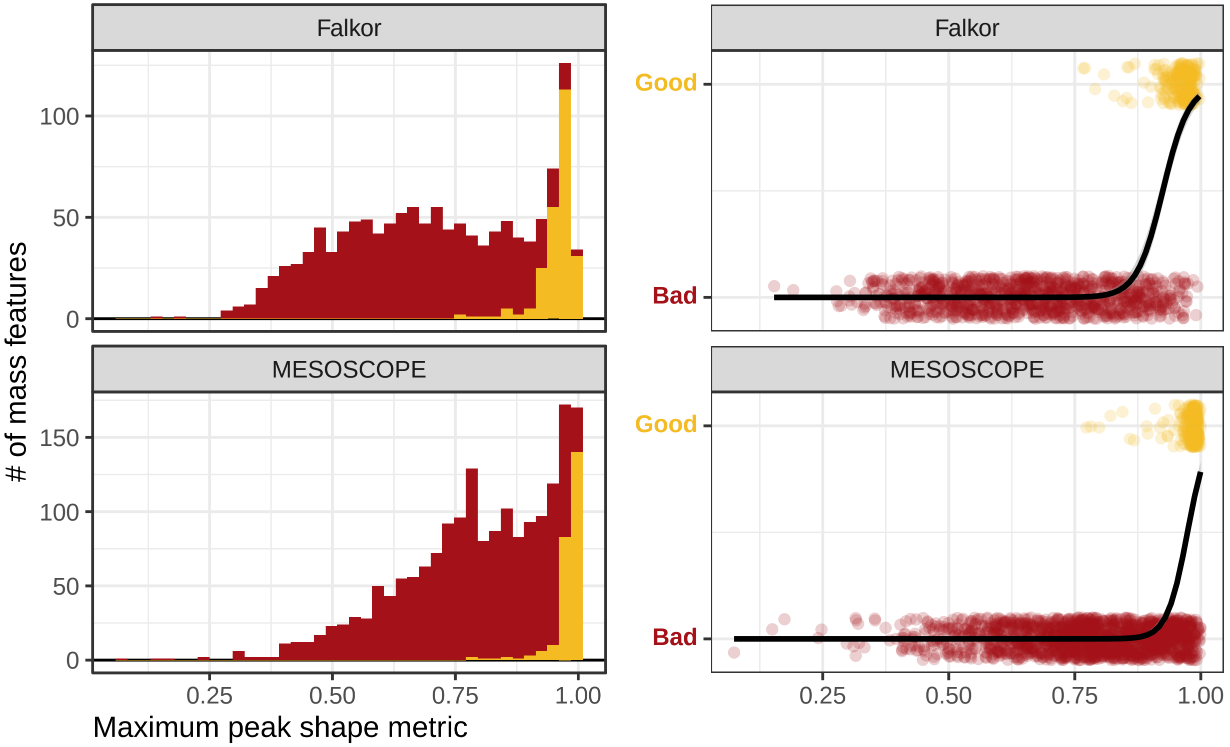

Falkor
MESOSCOPE
Falkor
MESOSCOPE
# of mass features
Maximum peak shape metric
Good
Bad
0
50
100
150
0.25
0.50
0.75
1.00

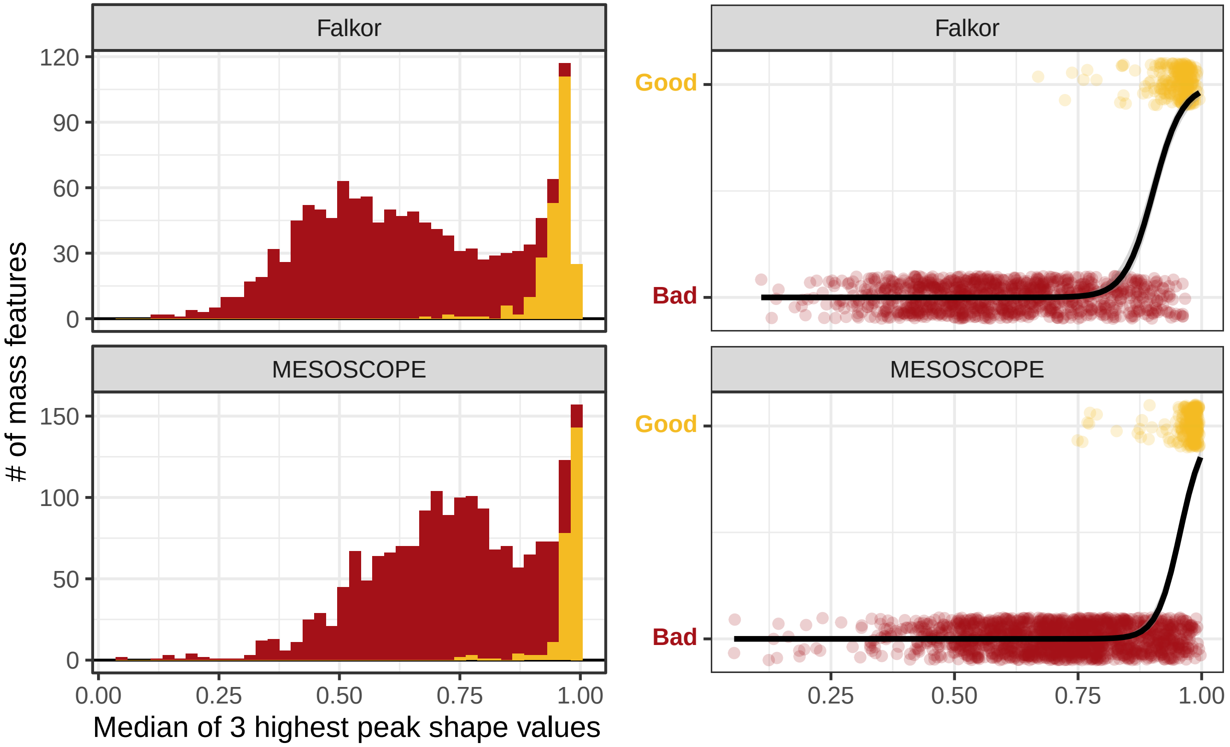

Falkor
120
90
60
30
0
MESOSCOPE
150
100
50
0
0.00
0.25
0.50
0.75
1.00
# of mass features
Median of 3 highest peak shape values
Falkor
Good
Bad
MESOSCOPE
Good
Bad
0.25
0.50
0.75
1.00

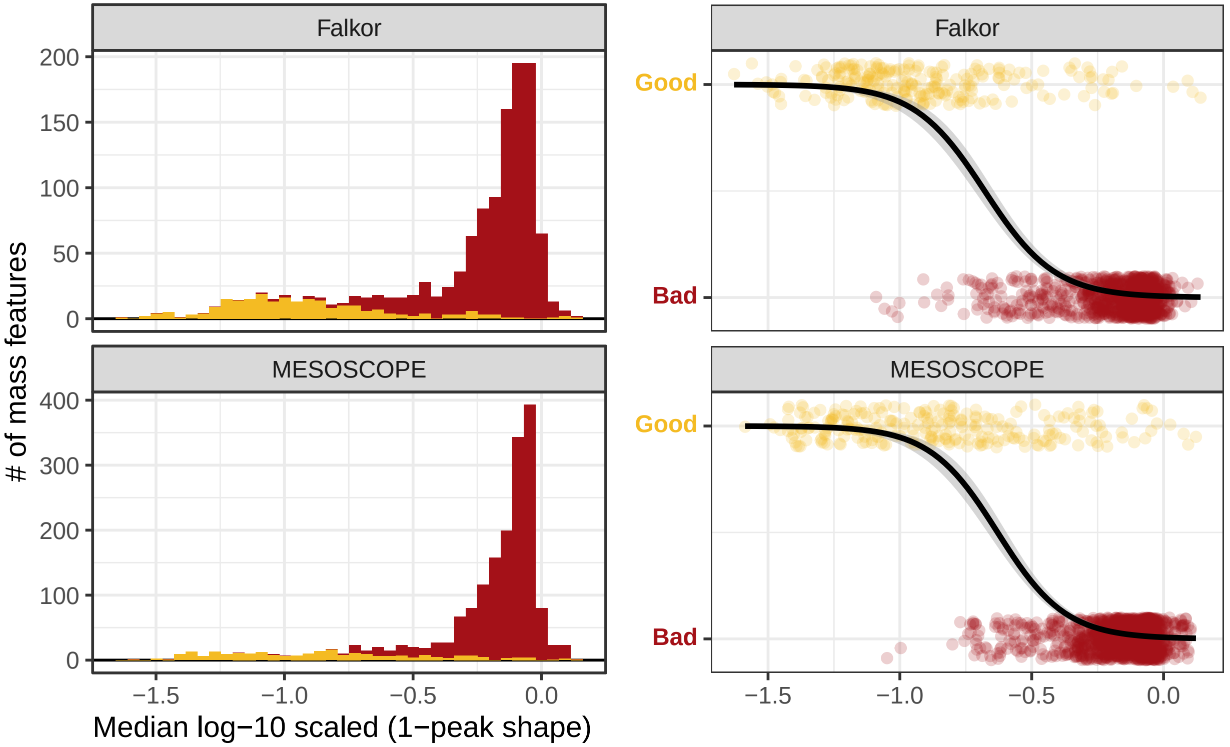

Falkor
MESOSCOPE
Falkor
MESOSCOPE
# of mass features
200
150
100
50
0
400
300
200
100
0
−1.5
−1.0
−0.5
0.0
Median log−10 scaled (1−peak shape)
Good
Bad
Good
Bad

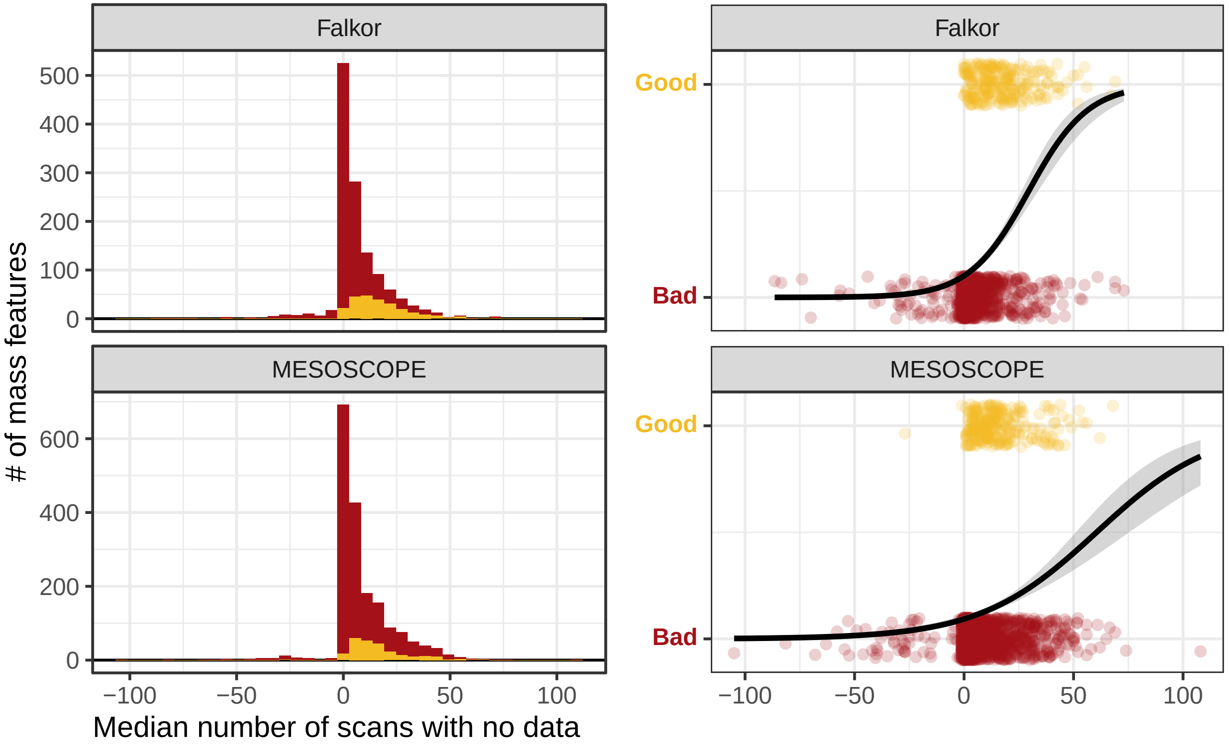
Falkor
MESOSCOPE
# of mass features
500
400
300
200
100
0
600
400
200
0
−100
−50
0
50
100
Median number of scans with no data
Good
Bad
Falkor
MESOSCOPE
Good
Bad
−100
−50
0
50
100

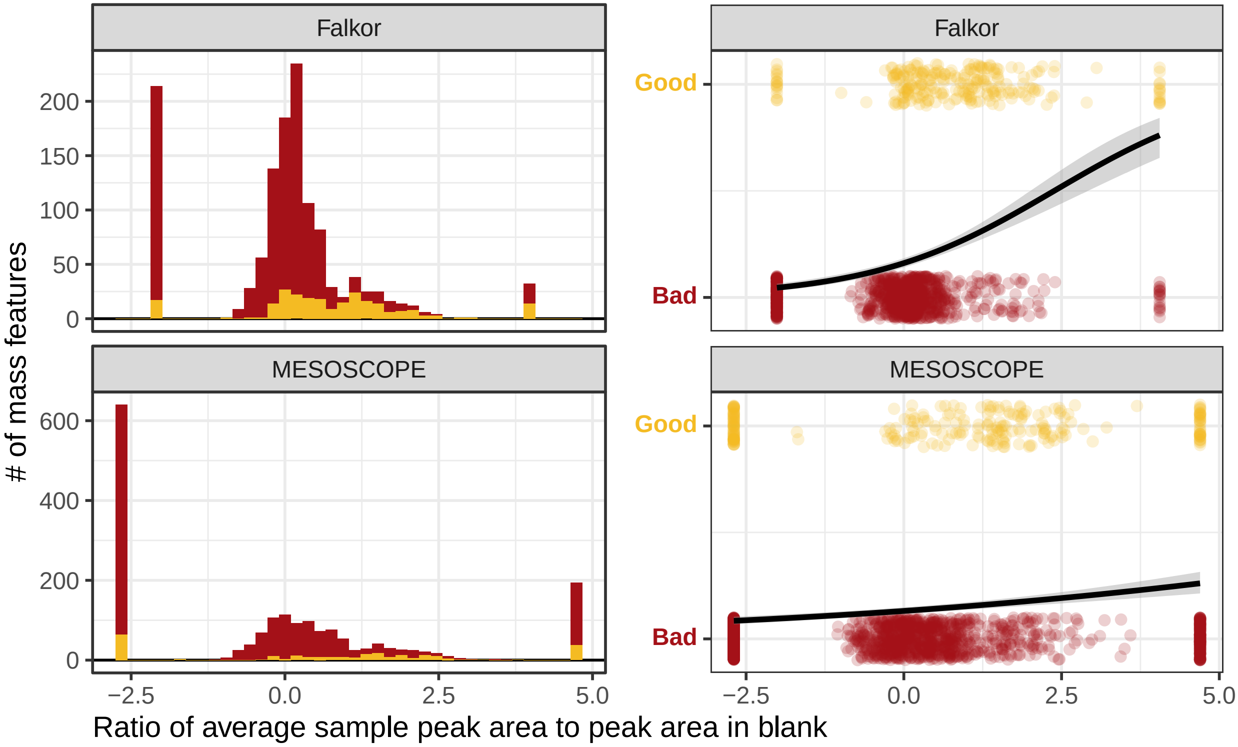

Falkor
MESOSCOPE
Falkor
MESOSCOPE
# of mass features
0
50
100
150
200
0
200
400
600
Good
Bad
Good
Bad
−2.5
0.0
2.5
5.0
−2.5
0.0
2.5
5.0
Ratio of average sample peak area to peak area in blank

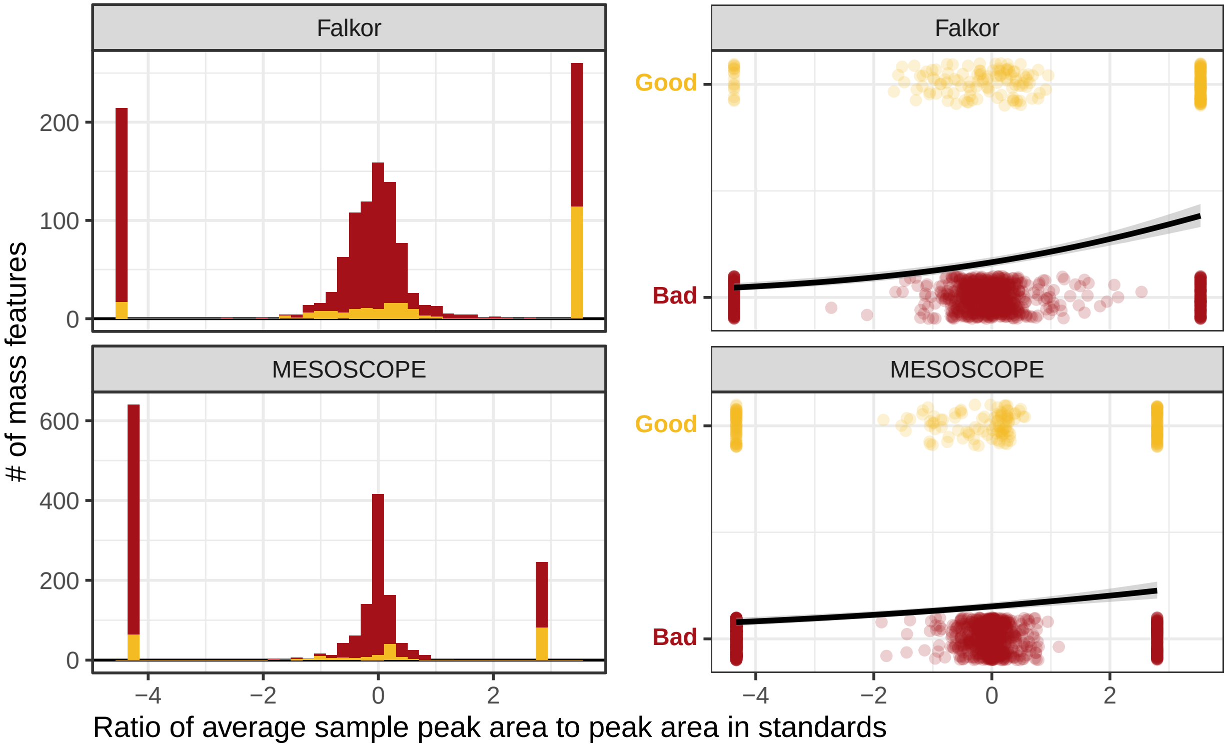
Falkor
Falkor
MESOSCOPE
MESOSCOPE
Good
Bad
Good
Bad
200
100
0
600
400
200
0
−4
−2
0
2
−4
−2
0
2
# of mass features
Ratio of average sample peak area to peak area in standards

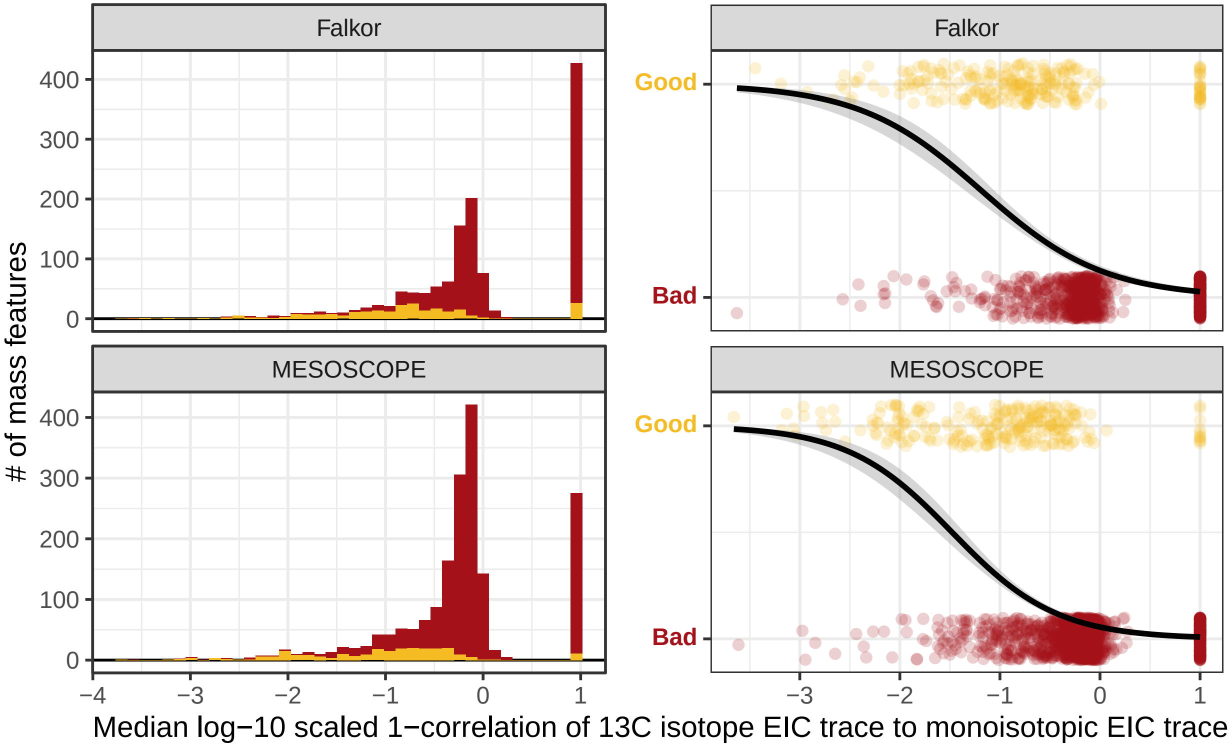
Falkor
Falkor
Good
Bad
MESOSCOPE
MESOSCOPE
Good
Bad
# of mass features
400
300
200
100
0
−4
−3
−2
−1
0
1
Median log−10 scaled 1−correlation of 13C isotope EIC trace to monoisotopic EIC trace

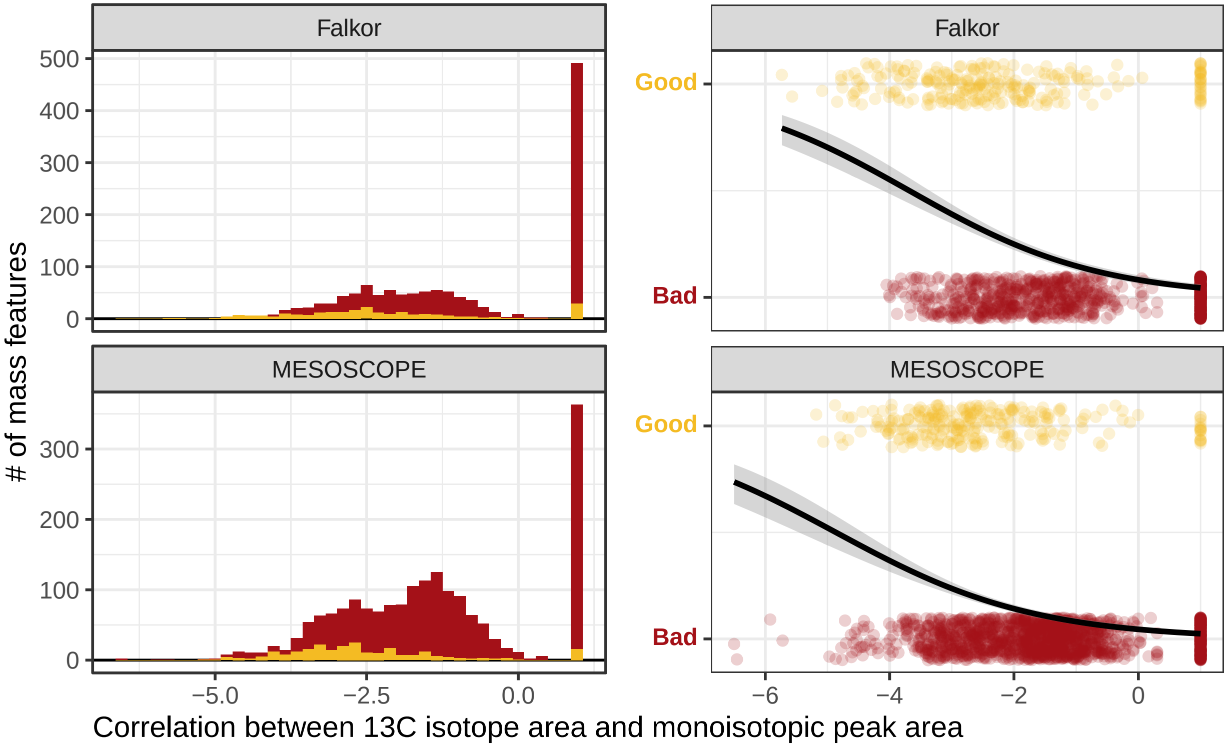
Falkor
Falkor
MESOSCOPE
MESOSCOPE
Good
Bad
Good
Bad
500
400
300
200
100
0
300
200
100
0
−5.0
−2.5
0.0
−6
−4
−2
0
# of mass features
Correlation between 13C isotope area and monoisotopic peak area
