# Supplementary figures and images for: Picky with peakpicking: assessing chromatographic peak quality with simple metrics in metabolomics
Source: BMC Bioinformatics. 2023 Oct 28;24:404. doi: 10.1186/s12859-023-05533-4 (PMC10612323; doi:10.1186/s12859-023-05533-4)

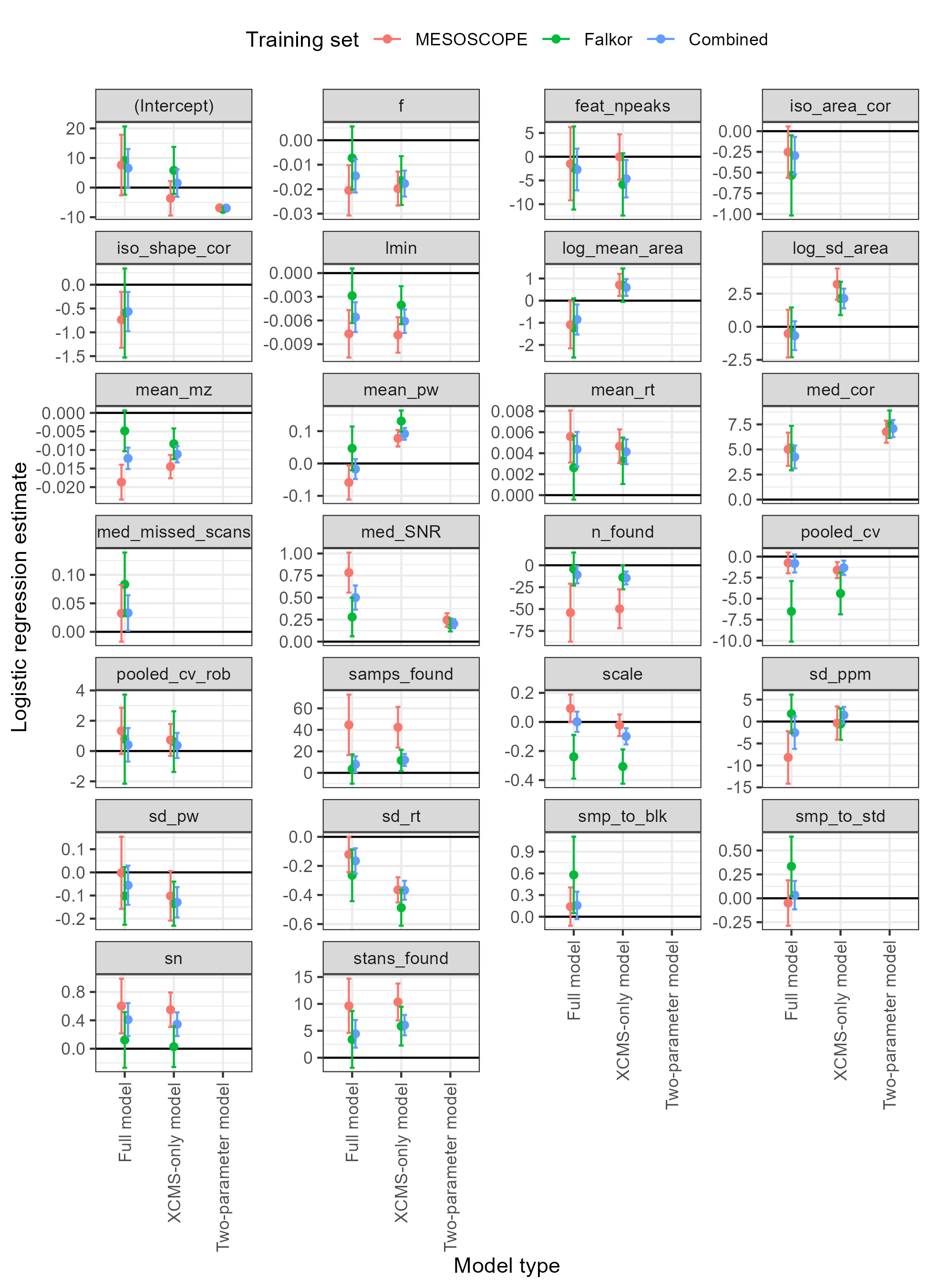

Supplement: Supplementary file 3 — Additional file 3: Figure S2 Model parameter estimates for each of the metrics in the full model, additionally broken down by their inclusion in the two-parameter (raw_data) and XCMS-exclusively models. Colors correspond to the dataset used to train the logistic regression model, with "both" indicating a combined model using all manually-labeled features across both datasets. [file 12859_2023_5533_MOESM3_ESM.png]

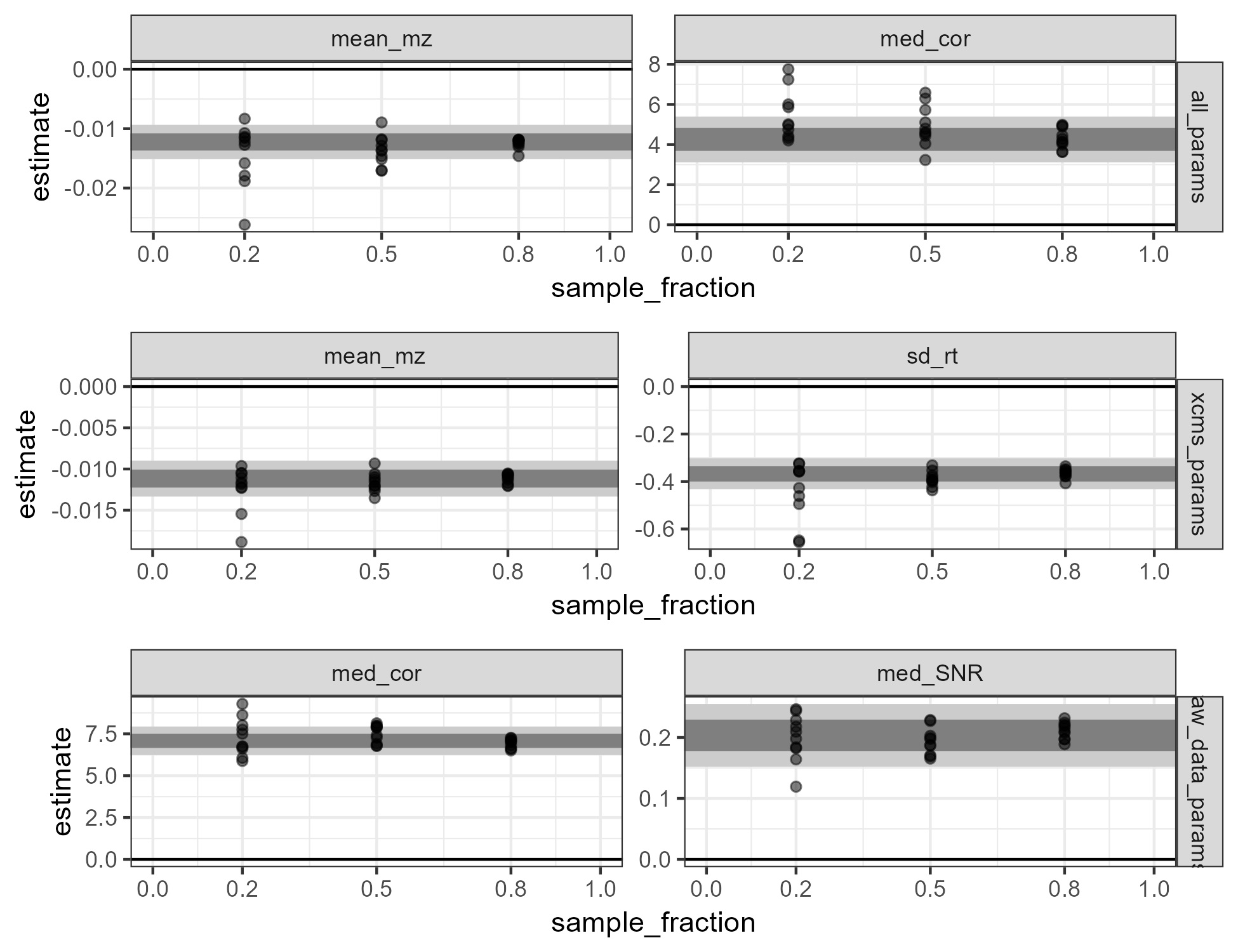

Supplement: Supplementary file 4 — Additional file 4: Figure S3 Robustness of the two most significant metrics across the full (all_params), XCMS-only (xcms_params), and two-parameter (raw_data_params) models. The x-axis corresponds to the fraction of the data used to train the model and the y-coordinate shows the estimated value for the specified term in the subset across tenfold replicated subsampling. The grey bar in the background corresponds to the estimate of the full model ± 1SE (thinner dark grey bar) and 2SE (thicker light grey bar). [file 12859_2023_5533_MOESM4_ESM.png]

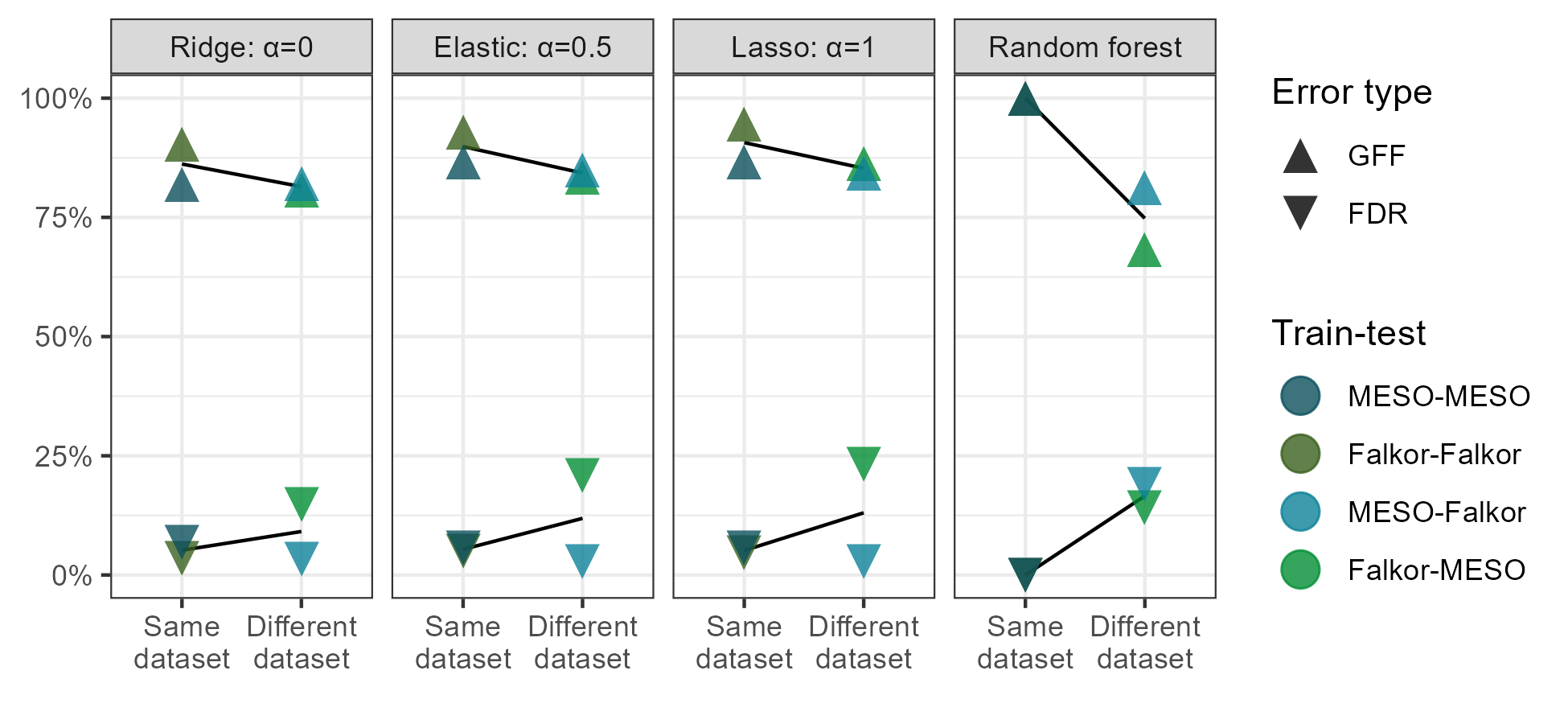

Supplement: Supplementary file 5 — Additional file 5: Figure S4 Performance of regularized regression and random forest models on internally (same train-test) and externally (different train-test) validated datasets. [file 12859_2023_5533_MOESM5_ESM.png]
